# Acute effects of fine particulate matter (PM_2.5_) on hospital admissions for cardiovascular disease in Beijing, China: a time-series study

**DOI:** 10.1186/s12940-019-0506-2

**Published:** 2019-08-01

**Authors:** Endawoke Amsalu, Tianqi Wang, Haibin Li, Yue Liu, Anxin Wang, Xiangtong Liu, Lixin Tao, Yanxia Luo, Feng Zhang, Xinghua Yang, Xia Li, Wei Wang, Xiuhua Guo

**Affiliations:** 1 Department of Epidemiology and Health Statistics, School of Public Health, Capital Medical University, No.10 Xitoutiao, You’anmenWai, Fengtai District, Beijing, 100069 China; 2 Beijing Municipal Key Laboratory of Clinical Epidemiology, Capital Medical University, Beijing, China; 3 Beijing Municipal Commission of Health and Family Planning Information Center, Beijing, China; 4 Department of Mathematics and Statistics, La Trobe University, Melbourne, Victoria Australia; 5 Global Health and Genomics, School of Medical Sciences and Health, Edith Cowan University, Perth, Western Australia Australia

**Keywords:** Particulate matter, PM_2.5_, Admission, Cardiovascular diseases

## Abstract

**Background:**

Air pollution and cardiovascular disease are increasing problems in China. However, the short-term association between fine particulate matter (PM_2.5_) and cardiovascular disease (CVD) is not well documented. The purpose of this study is to estimate the short-term effects of PM_2.5_ on CVD admissions in Beijing, China.

**Methods:**

In total, 460,938 electronic hospitalization summary reports for CVD between 2013 and 2017 were obtained. A generalized additive model using a quasi-Poisson distribution was used to investigate the association between exposure to PM_2.5_ and hospitalizations for total and cause-specific CVD, including coronary heart disease (CHD), atrial fibrillation (AF), and heart failure (HF) after controlling for the season, the day of the week, public holidays, and weather conditions. A stratified analysis was also conducted for age (18–64 and ≥ 65 years), sex and season.

**Results:**

For every 10 μg/m^3^ increase in the PM_2.5_ concentration from the previous day to the current (lag 0–1) there was a significant increase in total CVD admissions (0.30, 95% CI: 0.20, 0.39%), with a strong association for older adults (aged ≥65 years), CHD (0.34, 95% CI: 0.22 to 0.45%) and AF (0.29, 95% CI, 0.03 to 0.55%). However, the observed increased risk was not statistically significant for HF hospitalizations. The associations in the single-pollutant models were robust to the inclusion of other pollutants in a two-pollutant model. No differences were found after stratification by sex and season.

**Conclusions:**

Exposure to PM_2.5_ increased the risk of hospitalizations from CVD, especially for CHD, and appeared to have more influence in the elderly. Precautions and protective measures and efforts to reduce exposure to PM_2.5_ should be strengthened, especially for the elderly.

**Electronic supplementary material:**

The online version of this article (10.1186/s12940-019-0506-2) contains supplementary material, which is available to authorized users.

## Introduction

Despite considerable progress in the reduction of air pollution and its corresponding impact on health, air pollution studies have attracted more attention [1]. PM_2.5_ is the most sensitive marker of air pollution and environmental risk factors [2]. The effect of PM_2.5_ on CVD risk has been widely reported by both short- and long-term epidemiological studies [3, 4]. However, there is limited evidence from countries where severe environmental pollution and cardiovascular morbidity and mortality have become a challenge, particularly in Asian developing countries [5].

In China, 27% of cities experience extreme air pollution, and morbidity and mortality from CVD have been rising [6]. Several studies associate an increase in CVD morbidity and mortality with PM_2.5_ air pollution [7–9]. However, most of these studies mainly focus on emergency room visits and cause-specific CVD mortality [10–12]. For instance, a multicity study in China showed that a 10 μg/m^3^ increase of PM_2.5_ was associated with an increase in daily cardiovascular disease mortality of 0.315% (95% CI: 0.133–0.497%) [13]. Although the use of hospital admissions is a more sensitive indicator than mortality [14] and has great public health importance, adverse short-term effects of PM_2.5_ on cause-specific CVD hospital admissions are not well documented in large cities such as Beijing. Moreover, the impact of PM_2.5_ on the city-specific level has not been comprehensively reported. In this study, we estimate the daily effect of PM_2.5_ on admissions for CVD and its subtypes (coronary heart disease: CHD, heart failure: HF, and atrial fibrillation: AF) in Beijing using a single-pollutant model, a two-pollutant model, and several subgroup analyses.

## Methods

### Cardiovascular data

Records from hospital admissions for CVD between 1 January 2013 and 31 December 2017 were extracted from the Beijing Public Health Information Center (http://www.phic.org.cn/). We extracted information on the patient’s date of hospital admission, principal diagnosis, age, and sex from each hospital admission record. Cause-specific CVD hospitalizations were identified based on the International Classification of Diseases, 10th Revision (ICD-10) codes: CHD (ICD-10: I20-I25), AF (ICD-10: I48) and HF (ICD-10: I50). In this study, the total number of CVD admissions was calculated as the sum the number of CHD, AF and HF admissions. Hospital admissions for CVD for patients under 18 years old were excluded from the current analysis because of the small number of records.

We did not use individual data identifiers; therefore, informed consent was not specifically required, but an official permit was required to access the data. The Institutional Review Board of Capital Medical University approved the study protocol (IRB00009511).

### Air pollution and meteorological data

Air pollution data were obtained from 35 fixed-site air quality–monitoring stations from Beijing Municipal Environmental Protection Bureau (http://www.bjepb.gov.cn/) between 1 January and 31 December 2017, covering nearly every district (at the county level) in Beijing. The 24-h average concentrations of five pollutants were used in this study: particulate matter with an aerodynamic diameter less than 2.5 μm (PM_2.5_), carbon monoxide (CO), sulfur dioxide (SO_2_), nitrogen dioxide (NO_2_) and the daily maximum 8-h average ozone concentrations (O_3_). In addition, the mean air pressure, the daily mean temperature and the mean relative humidity were extracted for the same study period from the China Meteorological Data Sharing Service System (https://data.cma.cn/en).

### Study design

An ecological time series design was conducted to estimate the association between the short-term effects of PM_2.5_ and hospital admissions for CVD. A time-series analysis based on general additive models have been widely used in epidemiological studies of air pollution to explore the short-term effects of air pollutant exposure on the risk of acute events.

### Statistical analysis

A generalized additive model using a quasi-Poisson distribution was applied to estimate the effect of PM_2.5_ on hospital admissions for CVD. The core model adjusted for the season, public holidays, the day of the week (DOW), and a long-term trend was created. A spline S (.) with 7 degrees of freedom (*df*) per year for a given time period was used to control for seasons and long-term trends, and 3 degrees of freedom (*df*) were used for temperature and relative humidity. DOW was used as a categorical variable, and public holidays were included as a two-level factor. The degrees of freedom (*df*) for calendar time, temperature and relative humidity were selected based on the parameters used in previous studies and were further tested by sensitivity analyses. A nonlinearity test using smoothing splines and 3 *df* graphically described the relationship (lag 0–1). A delayed-effect association was analyzed with separate lag structures for single day lags (from lag 0 to lag 3) and multiday lags (lag 0–1 and lag 0–3). We calculated the percentage change from the relative risk and Z-value to test the statistical significance of each subgroup difference with the formula $\mathrm{Z}=\left({\beta}_1-{\beta}_2\right)/\sqrt{SE_1^2+{SE}_2^2}$, where *β*_1_ and *β*_2_ are the effect estimates for the two categories, and *SE*_1_ and *SE*_1_ are their respective corresponding standard errors [10]. We used a two-step model. First, the model that included the main single pollutant (PM_2.5_) was entered alone. Second, two-pollutant models for SO_2_, CO, O_3_, and NO_2_ were created, and the effects were estimated. Subgroup analyses by sex, age, and seasonal variation were also performed. We mainly reported the effect of PM_2.5_ using a 2-day moving average concentration (lag 0–1) because this lag was more strongly associated with health effects. Additionally, to avoid the likely bias of the estimate of the effect PM_2.5_ due to an inadequate control of temporal trends, we also performed an analysis by period to evaluate possible temporal trends in the health effects. Therefore, we conducted an analysis for each year and reported the percentage change with 95% confidence intervals for a 10 μg/m^3^ increase in PM_2.5_. All data analyses were conducted with R version 3.0.1 (R Development Core Team, 2013).

### Sensitivity analysis

We conducted a series of sensitivity analyses by using alternative degrees of freedom (df) for calendar time, temperature and humidity. We used 5–10 df per year for time and 3–10 df for temperature and humidity [15].

## Results

In total, 460,938 hospital admissions from CVD were reported during the 5-year study period in Beijing, including 378,090 CHD, 24,455 AF and 58,393 HF admissions. Of these admissions, 54.9% were men, and 37.6% were under 65 years of age (Additional file 1). Table 1 shows the statistical descriptions of the daily hospital admissions for CVD, air pollution concentrations, and weather conditions. There were 252 hospital admissions from CVD per day on average.

**Table 1:** Statistical descriptions of cardiovascular admissions, atmospheric pollutants, and meteorological variables during the study period (2013–2017)

	Minimum	P_25_	Median	Mean (SD)	P_75_	Maximum
Hospital admission						
Cardiovascular disease	9	106	192	252.4 (165.1)	405	749
Coronary heart disease	2	77	159	207.1 (141.8)	340	632
Atrial fibrillation	0	4	10	13.4 (10.5)	22	53
Heart failure	0	20	29	32.0 (15.5)	43	90
Atmospheric pollutants						
SO_2_ (μg/m^3^)	0	4	8	15.31 (18.31)	19	133
NO_2_ (μg/m^3^)	0	34	44.	49.69 (23.34)	61	155
O_3_ (μg/m^3^)	0	49	81	95.66 (63.039)	136	367
PM_10_ (μg/m^3^)	0	50	86	102.30 (75.88)	130	820
PM_2.5_ (μg/m^3^)	0	30	59	76.86 (66.38)	102	477
Meteorological factors						
Average Temperature (°C)	−16.92	0.99	14.52	13.09 (12.34)	24.09	38.51
Maximum Temperature (°C)	−13.41	11.88	24.60	24.62 (15.80)	35.83	59.89
Minimum Temperature (°C)	−19.16	−3.88	6.98	6.16 (12.08)	17.06	30.94
Humidity (%)	1.121	5.24	7.47	24.38 (26.66)	43.30	95.30
Air pressure (hPa)	970.5	985.50	993.0	993.90 (10.51)	1001.5	1022.40

P_25_ = 25th percentile. P_75_ = 75th percentile. CO = carbon monoxide. PM_2.5_ = particulate matter with an aerodynamic diameter less than 2.5 μm. SO_2_ = sulfur dioxide. NO_2_ = nitrogen dioxide. O_3_ = ozone

During the study period, the mean daily pollution concentration was 76.9 μg/m^3^ for PM_2.5_, 15.3 μg/m^3^ for SO_2_, and 49.7 μg/m^3^ for NO_2_, and the mean 8-h maximum concentration for O_3_ was 95.7 μg/m^3^. The daily mean ambient temperature was 13.9 °C, the relative humidity was 24.4%, and the air pressure was 1016.7 hPa (Table 1).

**Fig. 1:**
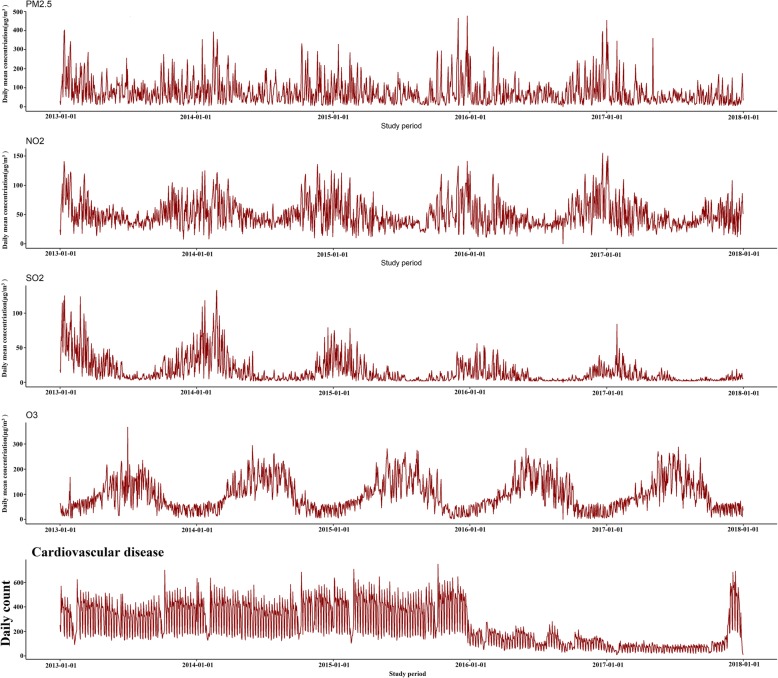
Time series plot of atmospheric pollutants and total CVD admissions from 2013 to 2017

To visualize seasonal and long-term trends, we plotted the CVD admissions, atmospheric pollutants and meteorological factors against time using time-series graphs (Fig. 1).

Table 2 shows Spearman’s correlations for different air pollution concentrations and weather conditions during the study period. There were strong-to-moderate positive correlations between PM_2.5_ and CO (*r* = 0.724), NO_2_ (*r* = 0.722) and PM_10_ (*r* = 0.841), but the correlation between PM_2.5_ and O_3_ was negative and weak (*r* = − 0.1127).

**Table 2:** Spearman’s correlations between each atmospheric pollutants and meteorological factors in Beijing, 2013-2017^b^

Variables	SO_2_	CO	NO_2_	O_3_	PM_10_	PM_2.5_	Temp	Humidity
SO_2_	1.000							
CO	0.6039	1.0000						
NO_2_	0.6552	0.7221	1.0000					
O_3_	−0.3621	− 0.4185	−0.4062	1.0000				
PM_10_	0.5715	0.5783	0.7005	−0.0304	1.0000			
PM_2.5_	0.5607	0.7246	0.7170	−0.1127	0.8417	1.0000		
Temp	−0.4938	− 0.3407	− 0.3014	0.8102	− 0.0197	− 0.0588	1.0000	
Humidity	−0.3893	0.0429	−0.0118	0.0108	−0.0279	0.0779	0.1052	1.0000

b All correlation coefficients were statistically significant (*P* < 0.001)

Temp: Temperature

**Fig. 2:**
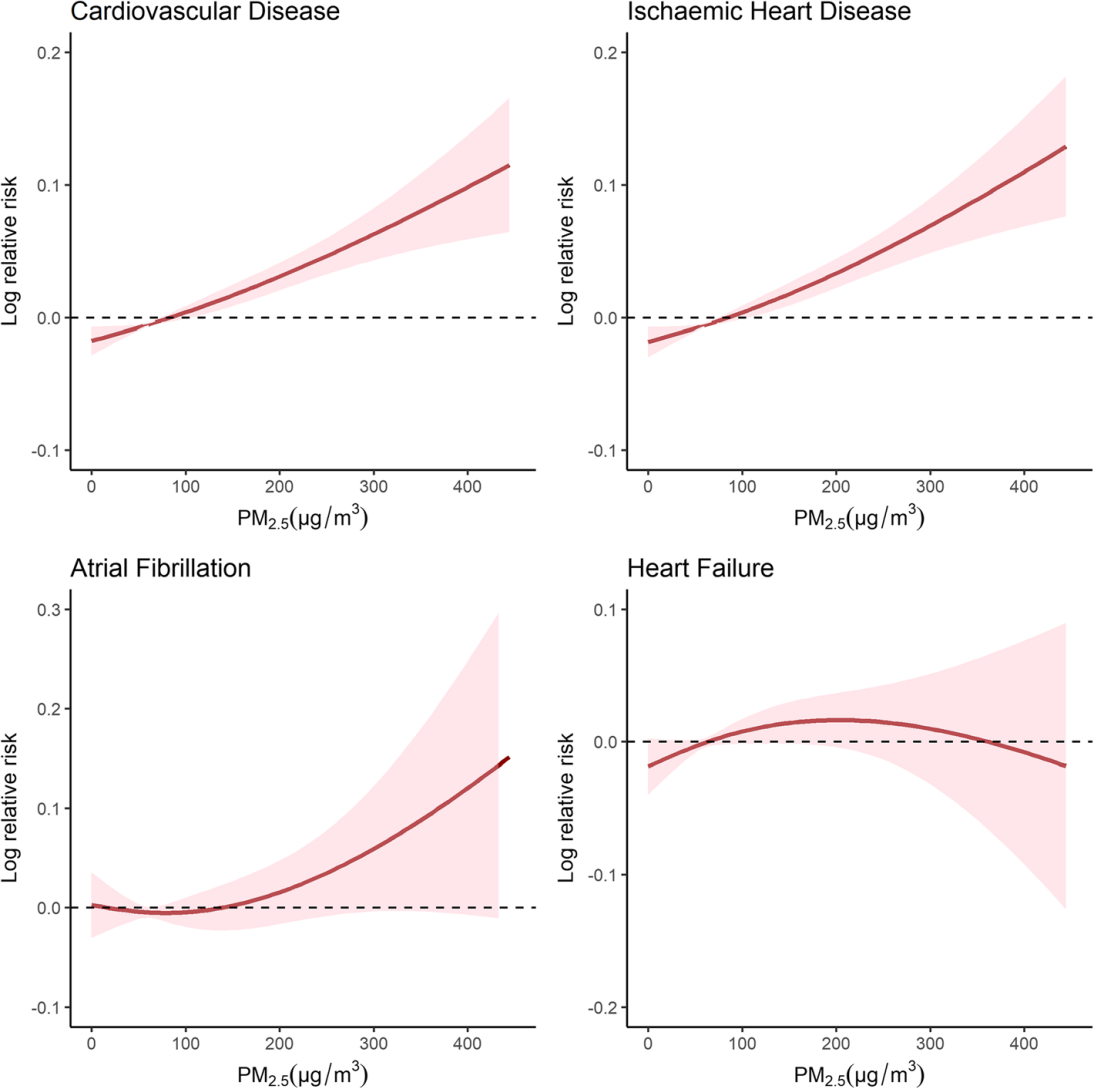
Exposure-response relationship curves for the association between hospital admissions for total and cause-specific cardiovascular disease and the 2-day moving average (lag 0–1) of PM_2.5_ concentrations

Fig. 2 shows the exposure-response relationship curve of the PM_2.5_ concentrations with total and cause-specific CVD admissions at lag0–1. The estimated dose-response relationships of the PM_2.5_ concentrations with total CVD and CHD admissions showed a linear relationship with a sharp increase in the dose-response function at lower concentrations (0–50 μg/m^3^) and a moderate increase at higher concentrations. For HF, the curve tended to plateau at higher PM_2.5_ concentrations (200 μg/m^3^). For AF, there appeared to be a small increase in the risk until the PM_2.5_ concentration exceeded approximately 190 μg/m^3^.

**Fig. 3:**
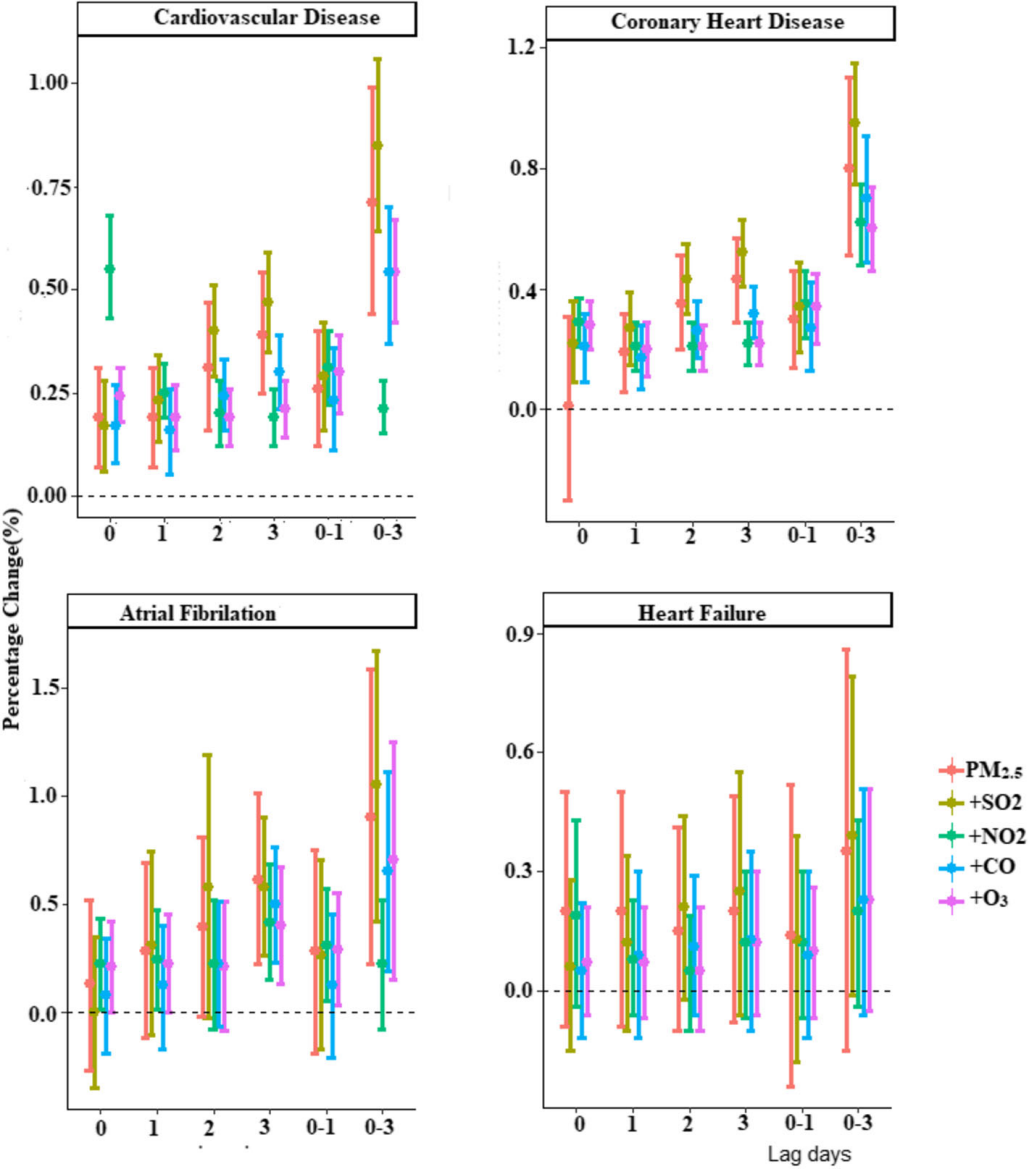
Percentage changes with 95% confidence intervals of hospital admissions for total and cause-specific cardiovascular disease associated with a 10 μg/m^3^ increase in daily PM_2.5_ concentrations with varying lag patterns

Fig. 3 shows the effect of PM_2.5_ (per 10 μg/m^3^ increase) with 95% CIs for daily hospital admissions for CVD, CHD, HF and AF for single and cumulative lag days. We found evidence for significant positive associations for at least one exposure lag structure between day-to-day variations in the PM_2.5_ concentration and hospital admissions for all cardiovascular outcomes, except for HF. The largest effect was observed at lag 3 for the single-day effect and at lag 0–3 for the cumulative day effect for total CVD, CHD and AF. A 10 μg/m^3^ increase from the previous day to the current day (lag0–1) in the single-pollutant model was associated with significant increases in hospital admissions for CVD (0.30, 95% CI: 0.20, 0.39%), CHD (0.34, 95% CI: 0.22, 0.45%), and AF (0.29, 95% CI: 0.03, 0.55%). No significant association was observed for HF (all *P* > 0.05).

Table 3 shows the two-pollutant model for the effects of PM_2.5_ at the current and previous lag day (lag 0–1) effect. The observed associations in the single-pollutant models were robust to the inclusion of the copollutants in the two-pollutant model. For CVD, the observed associations in the single pollutant model were robust but were attenuated with the inclusion of all copollutants except for O_3_ (0.31, 95% CI: 0.22, 0.40%). For CHD, the observed associations in the single-pollutant model were robust to the inclusion of copollutants and the association with NO_2_ was slightly increased (0.95, 95% CI: 0.75, 1.15%). For AF, the effect of PM_2.5_ was eliminated after adjusting for other copollutants, except for O_3_. However, the model remained trivial for HF after controlling for all pollutants (Table 3).

**Table 3:** Percentage changes with 95% confidence intervals for the increase in daily cardiovascular admissions with a 2-day moving average (lag 0–1) based on particulate matter (PM_2.5_) concentrations with and without adjustment for copollutants in Beijing, 2013–2017

Pollutants	CVD	AF	CHD	HF
Unadjusted PM_2.5_	0.30 (0.2,0.39)	0.29 (0.03,0.55)	0.34 (0.22,0.45)	0.10 (− 0.07,0.26)
Adjusted for SO_2_	0.23 (0.11,0.36)	0.12 (−0.21,0.45)	0.27 (0.13,0.42)	0.09 (− 0.12,0.3)
Adjusted for CO	0.26 (0.12–0.40)	0.28 (−0.19–0.75)	0.30 (0.14,0.46)	0.14 (− 0.24,0.52)
Adjusted for NO_2_	0.29 (0.16,0.42)	0.26 (−0.17,0.7)	0.95 (0.75,1.15)	0.13 (−0.18,0.39)
Adjusted for O_3_	0.31 (0.22,0.40)	0.31 (0.05,0.57)	0.35 (0.24,0.46)	0.11 (−0.05,0.27)

Data are percentage changes (%) and 95% confidence intervals

CO = carbon monoxide. PM_2.5_ = particulate matter with an aerodynamic diameter less than 2.5 μm. SO_2_ = sulfur dioxide. NO_2_ = nitrogen dioxide. O_3_ = ozone

CVD: Cardiovascular disease. CHD: Coronary Heart Disease

HF: Heart Failure. AF: Atrial Fibrillation

Table 4 presents the analysis for the period. For the whole period 2013–2017, we found evidence of significant positive associations at lag 0–1 between day-to-day variations in the PM_2.5_ concentration and hospital admissions for all cardiovascular outcomes. When models were analyzed for periods, the effect estimate for hospital admission become weaker and sharply decreased. A10 μg/m^3^ increase in PM_2.5_ exposure at lag 0–1 was associated with percent increase in hospital admissions for the total CVD (0.30, 95% CI; 0.20,0.39%) in 2013, (0.45, 95% CI; 0.30,0.60%) in 2014, (0.23, 95% CI;0.08,0.38%) in 2015, (− 0.30, 95%CI, − 0.69, 0.08%) in 2016 and (− 0.61, 95% CI, − 1.43, 0.22%) in 2017. A similar decline in effect estimate for cause-specific cardiovascular disease was also observed (Table 4).

**Table 4:** Percent change per 10 μg/m^3^ increase in PM_2.5_ for each year for cardiovascular hospital admission in Beijing, China

Year	Cardiovascular Disease	Coronary Heart Disease	Heart Failure	Atrial Fibrillation
2013				
Lag0	0.22(0.09,0.35)	0.23(0.09,0.37)	0.26(− 0.04,0.56)	− 0.24(− 0.7,0.23)
Lag1	0.19(0.11,0.27)	0.20(0.11,0.29)	0.34(0.2,0.66)	− 0.16(− 0.91,0.61)
Lag2	0.19(0.12,0.26)	0.21(0.13,0.28)	−0.01(− 0.34,0.32)	0.23(− 0.27,0.74)
Lag3	0.21(0.14,0.28)	0.22(0.15,0.29)	0.01(−0.35,0.37)	0.59(0.11,1.07)
Lag01	0.30(0.20,0.39)	0.12(−0.06,0.29)	0.43(− 0.05,0.80)	− 0.31(− 1.14,0.52)
Lag02	0.39(0.29,0.49)	0.10(− 0.12,0.32)	0.44(− 0.03,0.91)	− 0.17(− 1.17,0.84)
Lag03	0.54(0.42,0.67)	0.34(0.06,0.61)	0.52(− 0.06,1.10)	0.36(− 0.83,1.57)
2014				
Lag0	0.34(0.22,0.46)	0.38(0.25,0.50)	0.12(−0.18,0.41)	0.50(0.09,0.92)
Lag1	0.30(0.17,0.43)	0.33(0.19,0.47)	0.08(−0.21,0.38)	0.42(−0.03,0.88)
Lag2	0.27(0.14,0.40)	0.09(−0.14,0.22)	0.15(−0.17,0.48)	0.30(− 0.16,0.75)
Lag3	0.09(−0.30,0.21)	0.09(− 0.14,0.22)	0.15(− 0.17,0.48)	0.11(− 0.52,0.74)
Lag01	0.45(0.30,0.60)	0.50(0.34,0.66)	0.14(−0.2,0.48)	0.66(0.15,1.18)
Lag02	0.68(0.49,0.86)	0.76(0.55,0.96)	0.26(−0.17,0.70)	0.9(0.25,1.56)
Lag03	0.84(0.60,1.07)	0.93(0.68,1.18)	0.36(−0.17,0.90)	1.26(0.45,2.07)
2015				
Lag0	0.11(−0.02,0.24)	0.11(−0.03,0.26)	0.11(− 0.15,0.37)	0.29(− 0.09,0.67)
Lag1	0.23(0.10,0.36	0.23(0.09,0.37)	0.18(−0.1,0.46)	0.39(−0.02,0.80)
Lag2	0.10(−0.04,0.24)	0.11(−0.06,0.28)	− 0.03(− 0.31,0.26)	0(−0.51,0.50)
Lag3	0.14(0.02,0.27)	0.14(0.01,0.27)	0.08(−0.29,0.45)	0.31(−0.08,0.70)
Lag01	0.23(0.08,0.38)	0.24(0.07,0.40)	0.19(−0.13,0.51)	0.47(0,0.95)
Lag02	0.29(0.11,0.47)	0.30(0.01,0.50)	0.17(−0.22,0.56)	0.46(−0.12,1.03)
Lag03	0.40(0.19,0.61)	0.42(0.09,0.64)	0.18(−0.28,0.64)	0.76(−0.06,1.58)
2016				
Lag0	0.07(00.3, 0.17)	−0.12(− 0.40,0.13)	0.27(− 0.14,0.69)	−0.21(−1.50,1.09)
Lag1	−0.45(− 0.81,-0.09)	−0.52(− 1.00,-0.13)	−0.19(− 0.86,0.48)	−1.44(−2.35,-0.53)
Lag2	−0.35(− 0.7,-0.01)	−0.56(− 1.00,-0.13)	−0.03(− 0.47,0.54)	−0.24(− 1.21,0.74)
Lag3	− 0.05(− 0.30,0.20)	−0.035(− 0.30,0.23)	0.08(− 0.45,0.61)	−0.25(− 1.15,0.66)
Lag01	− 0.3(− 0.69,0.08)	−0.38(− 0.79,0.03)	0.09(− 0.5,0.68)	−1.44(− 2.56,-0.32)
Lag02	−0.54(− 1.04,-0.03)	−0.72(− 1.25,-0.20)	0.12(− 0.65,0.89)	−1.74(− 2.99,-0.69)
Lag03	−0.67(− 1.31,-0.03)	−0.90(− 1.56,-0.23)	0.17(− 0.65,1.00)	− 2.36(−4.01,-0.69)
2017				
Lag0	− 0.66(− 1.04,-0.27)	−0.56(− 1.01,-0.11)	−0.58(− 1.26,0.10)	−0.79(− 2.94,1.39)
Lag1	− 0.0013(− 0.75,0.50)	−0.53(− 1.05,-0.01)	−0.13(− 0.75,0.50)	−0.14(− 1.72,1.48)
Lag2	− 0.025(− 1.15,1.10)	−0.16(− 2.09,1.77)	−0.04(− 0.15,0.07)	−1.08(− 1.35,-0.81)
Lag3	−0.96(− 1.39,1.54)	−0.06(− 1.73,1.60)	−0.09(− 0.39,0.21)	−1.42(− 1.69,-0.57)
Lag01	−0.66(− 1.43,0.10)	−0.84(− 1.46,-0.21)	−0.61(− 1.43,0.22)	−1.01(− 3.39,1.37)
Lag02	− 0.28(− 1.21,0.66)	−0.01(− 0.94,0.92)	−0.13(− 1.21,0.66)	−0.10(− 2.72,2.52)
Lag03	− 0.51(− 0.52,1.63)	−0.015(− 0.12,2.18)	−0.51(− 0.59,0.63)	−3.34(− 0.22,-6.46)

**Fig. 4:**
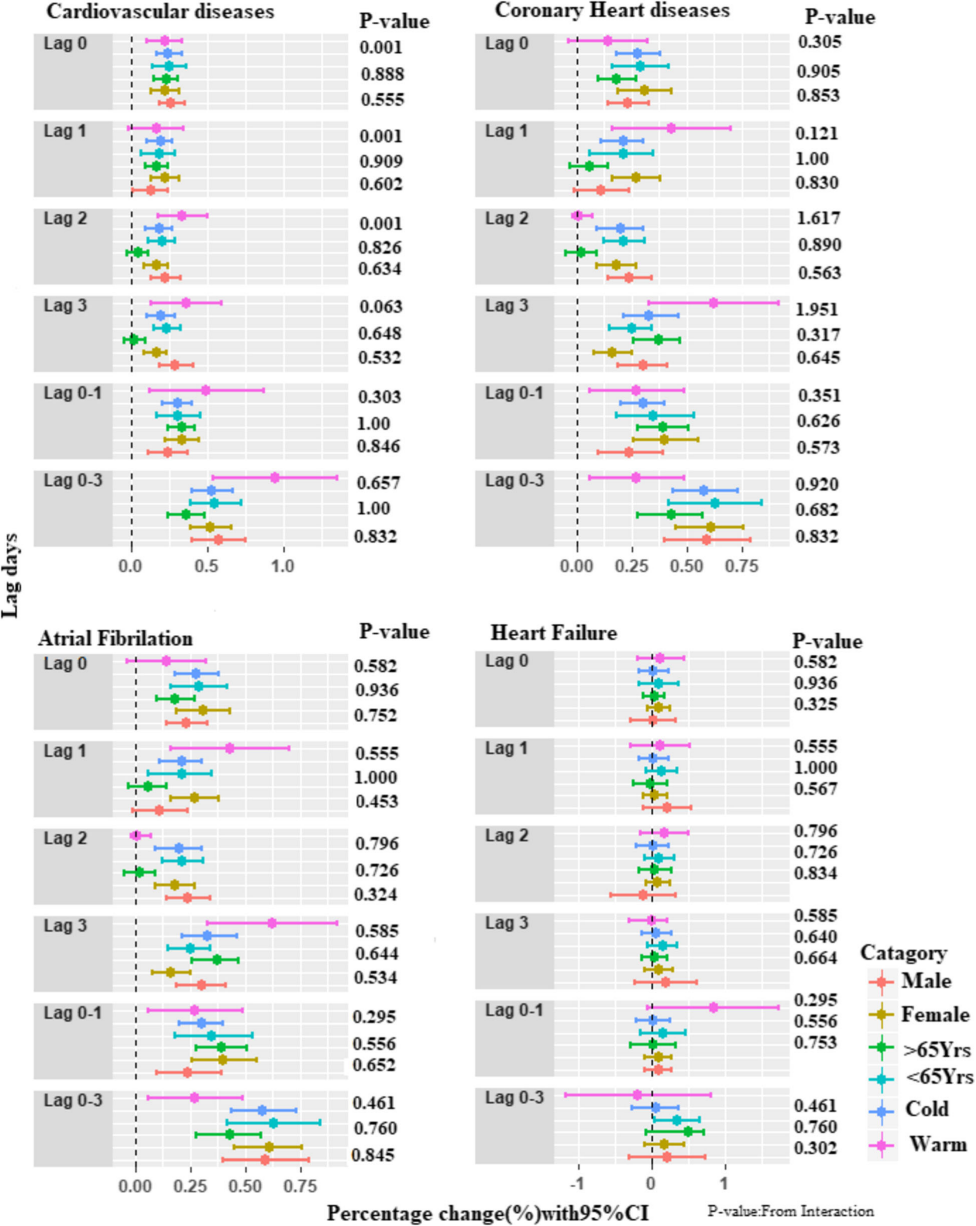
Percentage changes in daily hospital admissions for total and cause-specific cardiovascular disease for each 10 μg/m^3^ increase in the 2-day moving average (lag0–1) concentration of PM_2.5_, stratified by sex (male and female), season (cold and warm) and age (< 65 and ≥ 65 years)

Fig. 4 shows the associations between the PM_2.5_ concentrations and hospital admissions for CVD, CHD, AF and HF stratified by age group (lag 0–1 days). We observed a significant difference between age groups at a moving average of lag0–1_,_ with an interaction *P* = 0.001, for total CVD. No significant difference was observed after stratifying by sex and season.

The effect estimate of PM_2.5_ on CVD hospital admissions was relatively insensitive to the number of *df* specified for calendar time, for smoothing of temperature and for relative humidity (see Additional file 1: Table S1), suggesting that our core model is relatively robust to model specification.

## Discussion

In the present study, a time-series analysis based on a generative additive Poisson model was used to investigate the associations between PM_2.5_ and hospitalizations for CVD in Beijing from 2013 to 2017. Short-term exposure to PM_2.5_ was found to be significantly associated with an increased risk of hospital admissions for CVD, CHD and AF, but not for HF. Robust findings were found after controlling for other pollutants in the two-pollutant models.

Previously, studies have shown positive associations between PM_2.5_ and CVD morbidity and mortality [7, 8, 13]. For example, a study conducted by Xu et al. found that a 10 μg/m^3^ increase in the PM_2.5_ concentration was associated with a 0.56% increase in CHD admissions at lag0–1 (95% CI: 0.16–0.95%), a 0.81% increase in heart rhythm disturbances (HRD) at lag0–1 (95% CI: 0.05–1.57%) and a 1.21% increase heart failure (HF) emergency room visits on the same day (95% CI: 0.27–2.15%) [16]. Chen C et al. conducted a multicounty time-series study demonstrated that a 10 μg/m^3^ increase in PM_2.5_ was associated with a 0.12% increase in cardiovascular disease (CVD) (95% CI, 0.001–0.25), a 0.42% increase in myocardial fraction (95% CI, 0.03–0.81), and a 0.17% increase in coronary heart disease (95% CI, − 0.04-0.40) [17]. A case-crossover study conducted with 12,865 patients in Utah, USA found that a 10 μg/m^3^ increase in PM_2.5_ concentration was associated with a 4.5% increase in the risk of acute ischemic coronary events (95% CI, 1.1 to 8.0%) [18]. A study conducted in Madrid, Spain, reported that a 10 μg/m^3^ increase in PM_2.5_ concentration on the same day was significantly associated with an 11.08%: (95% CI: 1.03, 1.13%) increase for hospital admissions due to circulatory causes [19]. Moreover, in different studies, the impact of PM_2.5_ on CVD is robust when adjusting for copollutants [16]. For example, a study conducted by Qu et al. found that the estimated effects of PM_2.5_ were robust after adjusting for SO_2_, O_3_, CO and NO_2_ [11]. Similarly, the association between PM_2.5_ concentration and ischemic stroke at lag 0 to 1 days was maintained after adjusting for other air pollutants (NO_2_, photochemical oxidants, or SO_2_) [20]. Our current findings were consistent with those of previous studies that explored the association between PM_2.5_ exposure and the risk of hospital admission for CVD after adjusting for CO, NO_2_, SO_2_ and O_3._

China, the largest developing country, has the highest ambient PM_2.5_ levels worldwide because of rapid urbanization and its energy consumption is the highest [1, 21]. The high level of urban PM_2.5_ in Chinese megacities (such as Beijing, Shanghai, Chongqing and Guangzhou) mainly originates from sources such as traffic-related emissions, road/soil dust, biomass burning, and agriculture activities as well as regional transported aerosols [22, 23], but research remains limited and the adverse health effects of PM_2.5_ on cardiovascular hospital admissions need to be quantified. Our findings were supported by a recent study conducted in Beijing, China that reported a 10 μg/m^3^ increase in PM_2.5_ concentrations on the same-day PM_2.5_ concentrations was associated with a 0.31% increase in the daily admissions for ischemic stroke (95% CI, 0.17–0.45%) [24]. A 10 μg/m^3^ increase in PM_2.5_ was associated with a 0.27% increase in CHD morbidity (95% CI 0.21 to 0.33%). Moreover, a per 10 μg/m^3^ increase in PM_2.5_ at lag 3 was associated with a 0.14% increase in cardiovascular visits (95% CI: 0.01–0.27%) [11]. Our results were consistent with previous studies performed in different Chinese cities that reported a significant PM_2.5_ exposure effect on lag 0 day and lag 1 day and a maximum PM_2.5_ exposure effect on lag 3 days.

Estimates of short term effect using time series studies are not confounded by factors that vary slowly over time. Based on various time series review, Bell Ml et al. concluded that the effect estimates for particulate matter and mortality are unlikely to be biased to a large degree by inadequate control for temporal trend [25]. However, proper adjustment for temporal trend is still a concern in today. In this study, we conducted an analysis by year for the effect estimate of PM_2.5_ to evaluate some possible temporal trends in hospitalization for CVD. We found a trend of decline in short-term effect of PM_2.5_ on hospitalization for CVD from 2013 to 2017. Our study provides the association between daily changes in PM_2.5_ levels and hospitalization is decreased sharply over time. A declining trend in the short-term risk estimates is evidence that the day-to-day association between PM_2.5_ and hospitalization from total and cause-specific CVD is getting weaker over time, possibly as a result of changes in the composition and toxicity of the PM_2.5_ from the air quality control programs [26, 27]. Moreover, a time trend of declining effect may be possibly as a result of greater exposure measurement error at lower levels of PM_2.5_; flattening of the exposure-response relation at lower concentrations of PM_2.5_; and a change in the underlying susceptibility of the population, a decline in smoking or reducing CVD rates [28, 29].

The exposure-response relationship assessment is crucial for public health policy as is the need for decision-making regarding the air pollution limit for PM_2.5_. A linear relationship was observed between PM_2.5_ levels and mortality due to diseases of the circulatory system in Madrid, Spain [30]. Nevertheless, the exposure-response relationship in severe air pollution environments such as Beijing remains unclear. In this study, we conducted an exposure-response relationship analysis to explore the pattern and scope of the adverse response. We observed an approximately linear exposure-response relationship, which is consistent with the recent study that explored the exposure-response relationship pattern for respiratory emergency visits related to PM_2.5_ [16]. Similarly, our study was supported by a study of 63,956 first hospital admissions for ischemic stroke, suggesting that the relationship was approximately linear, with a small fluctuation at lower concentrations (< 100 μg/m^3^) and a sharper increase at higher concentrations [24]. Moreover, a study involving 369,469 ischemic heart disease cases in Beijing suggested that PM_2.5_ at levels below 75 μg/m^3^ do not significantly increase the risk of ischemic heart disease, which is consistent with our study [31]. Based on these findings, we hypothesized that there might be a threshold concentration at which PM_2.5_ becomes harmful enough to impose an adverse impact on the development and progression of cardiovascular disease. Future studies are needed to clarify this important issue.

In this study, we also found that the association between short-term PM_2.5_ exposure and hospital admission varied by cause-specific CVD. The adverse effect was obvious and robust for daily hospitalizations for CHD and AF but not for HF. Adverse effects due to a short-term exposure to PM_2.5_ for CHD, AF and HF was also evident in other studies [18, 31, 32]. However, some inconsistent results have been reported for PM_2.5_ exposure for HF [33–36]. For example, *Poloniecki* et al. in London, United Kingdom, and *Symons* et al. in Baltimore, Maryland, USA, found no statistically significant associations between any pollutant and hospital admissions for HF [35, 36]. One explanation may be that HF is clinically heterogeneous and complicated by a large number of comorbid diseases that may result in outcome misclassification [37]. Similarly, misclassification of cardiovascular events was detected among the study participants in Baltimore, Maryland [36]. Furthermore, a study by *Dabass* et al. in the National Health and Nutrition Examination Survey (NHANES) confirmed no significant associations for either short-or long-term PM_2.5_ exposure with HF risk in the general adult population, but stronger associations were found among clinically heterogeneous and comorbid disease participants [38].

Increased vulnerability to PM_2.5_ health effects might be more common among more exposed populations [39]. Thus, consideration of the effect of PM_2.5_ on different these groups is crucial for public health policy. Some studies reported an increased risk of cardiovascular admissions in women and elderly people [31, 33, 34, 39]. In the present study, a difference in effect was found among elderly people (age ≥ 65) with a 0.52% increased risk (*P* = 0.001) for CVD hospitalization, but this effect was not present after stratifying by sex and season. Overall, our study found a more consistent and increased effect for CHD compared with AF and HF, which is supported by the 2010 evidence summary report from the American Heart Association (AHA) [39]. The short-term association of PM_2.5_ with CVD hospitalization is consistent with previous epidemiological studies, although the mechanisms of the PM_2.5_ effect remain unclear. However, different potential mechanisms have been proposed, such as oxidative stress, inflammation, elevation in stress hormones and metabolic alterations [39, 40]. After inhalation of PM_2.5_, a local inflammatory response is developed, and several proinflammatory mediators, such as IL-6 and TNF-α, are also increased, which induces an increase in the concentrations of blood fibrinogen and C-reactive protein (CRP), which are important markers of cardiovascular events. Numerous studies have demonstrated that exposure to particulate matter is associated with increased fibrinogen and CRP, resulting in an increased risk of CVD [41–43]. PM_2.5_ exposure can also disturb the autonomic nervous system (ANS) and results in heart rate variability (HRV), which is another potential mechanism for CVD [44]. Recent research demonstrates that PM_2.5_ also directly affects the cardiovascular system by entering into the systemic circulation and causing myocardial dysfunction through mechanisms of reactive oxygen species production, calcium ion interference, endothelial cell damage and vascular dysfunction [42, 45, 46].

This study has several strengths. First, cardiovascular hospital admissions data were obtained from an established monitoring system covering more than 172 comprehensive hospitals in Beijing, which resulted in a relatively large sample size. Second, compared with previous studies, a relatively larger sample size and recent data over a 5-year period were used, which allowed us to examine the associations at high levels of validity and reliability. Third, the inclusion of all 35 monitoring sites for air pollution better represents the effects of air pollution than other studies. However, this study also has limitations. Similar to other studies involving explorations of the impact of air pollutants on health outcomes, we need to carefully interpret and infer the causal relationship between PM_2.5_ exposure and hospitalizations for CVD due to the ecological design of the present study. Future epidemiological cohort studies are needed for the assessment of cause-specific cardiovascular disease, especially in elderly people.

## Conclusions

This study shows that short-term exposure to ambient PM_2.5_ significantly increased the risk of hospitalizations from total CVD, especially for CHD. Our results also provided evidence of the risk of air pollution due to PM_2.5_, which was relatively higher among older people. Precautions and protective measures and efforts to reduce exposure to PM_2.5_ should be strengthened, especially for elderly people.

## Additional file

Additional file 1: of Acute effects of fine particulate matter (PM_2.5_) on hospital admissions for cardiovascular disease in Beijing, China: A time-series study. (DOCX 19 kb)

## Data Availability

The data can be accessed from the Beijing Public Health Information Center with permission via direct request.

## Abbreviations

AF: Atrial Fibrillation
ANS: Autonomic Nervous System
CHD: Coronary Heart Disease
CRP: C-reactive protein
CVD: Cardiovascular Disease
HF: Heart Failure
HRV: Heart Rate Variability
WHO: World Health Organization
