# Supplementary material for: Acute effects of fine particulate matter (PM2.5) on hospital admissions for cardiovascular disease in Beijing, China: a time-series study
Source: Environ Health. 2019 Aug 1;18:70. doi: 10.1186/s12940-019-0506-2 (PMC6670159; doi:10.1186/s12940-019-0506-2)
Supplement: Supplementary file 1 — of Acute effects of fine particulate matter (PM2.5) on hospital admissions for cardiovascular disease in Beijing, China: A time-series study. (DOCX 19 kb) [file 12940_2019_506_MOESM1_ESM.docx]

| Table S1. Sociodemographic characteristics of total and cause-specific CVD admission in Beijing,China,2013-2017. | | | | | | | |
| --- | --- | --- | --- | --- | --- | --- | --- |
| Admission | Category | 2013 | 2014 | 2015 | 2016 | 2017 | Total |
|  |  | N(%) | N(%) | N(%) | N(%) | N(%) | N(%) |
| CVD | Male | 66174(56) | 69910(56) | 74946(56) | 23778(52) | 18340(51) | 253148(55) |
|  | Female | 52453(44) | 55861(44) | 59093(44) | 22095(48) | 18288(50) | 207790(45) |
|  | <65 | 72371(61) | 77377(61) | 81920(61) | 30781(67) | 25099(68) | 287548(62) |
|  | >65 | 46256(39) | 48394(39) | 52119(39) | 15092(33) | 11529(32) | 173390(38) |
| CHD | Male | 56978(57) | 59282(57) | 63204(57) | 18849(53) | 13876(52) | 212189(56) |
|  | Female | 43242(43) | 45288(43) | 47667(43) | 16648(47) | 13056(48) | 165901(44) |
|  | <65 | 57973(58) | 60873(58) | 64212(58) | 22365(63) | 16988(63) | 222411(59) |
|  | >65 | 42247(42) | 43697(42) | 46659(42) | 13132(37) | 9444(36) | 155679(41) |
| AF | Male | 2987(51) | 3483(51) | 4110(53) | 1152(49) | 813(47) | 12547(51) |
|  | Female | 2877(49) | 3209(49) | 3681(47) | 1226(51) | 915(53) | 11908(49) |
|  | <65 | 4054(69) | 4623(69) | 5244(67) | 1732(73) | 1240(72) | 16893(69) |
|  | >65 | 1810(69) | 2069(31) | 2547(33) | 648(27) | 488(28) | 7562(31) |
| HF | Male | 6209(49) | 7145(49) | 7632(49) | 3775(47) | 3651(46) | 28412(49) |
|  | Female | 6334(51) | 7364(51) | 7745(51) | 4221(53) | 4317(54) | 29981(51) |
|  | <65 | 10344(83) | 11881(83) | 12468(81) | 6684(84) | 6871(86) | 48244(83) |
|  | >65 | 2199(17) | 2628(17) | 2913(19) | 1312(16) | 1097(14) | 10149(17) |
| HF: Heart Failure, CVD: Cardiovascular disease, CHD: coronary heart disease, AF: atrial fibrillation | | | | | | | |

Supplementary material

Additional file:

**Article**: Acute effects of fine particulate matter (PM_2.5_) on hospital admissions for cardiovascular disease in Beijing, China: A time-series study

**Authors:** Endwoke Amsalu, Tianqi Wang_,_ Haibin Li, Yue Liu, Anxin Wang, Xiangtong Liu, Lixin Tao, Yanxia Luo, Feng Zhang, Xinghua Yang, Xia Li, Wei Wang, Xiuhua Guo

Table S1: Sociodemographic characteristics of total and cause-specific CVD admission in Beijing, China,2013-2017.

Table S2: Sensitivity analysis per 10 μg/m^3^ increase in PM_2.5_ at lag01 using different degree of freedom(*df*) for calendar time, temperature and relative humidity for total and cause-specific cardiovascular disease hospital admission in Beijing, China.

| Table S2: Sensitivity analysis per 10 μg/m^3^ increase in PM_2.5_ at lag01 using different degree of freedom(*df*) for calendar time, temperature and relative humidity for total and cause-specific cardiovascular disease hospital admission in Beijing, China | | |
| --- | --- | --- |
| Admission | Temperature & humidity | Calendar time |
| Cardiovascular disease |  |  |
| 3*df* | 0.29(0.20;0.38) |  |
| 4*df* | 0.31(0.22;0.40) |  |
| 5*df* | 0.34(0.26;0.43) |  |
| 6*df* | 0.36(0.27;0.44) | 0.28(0.18;0.37) |
| 7*df* | 0.35 (0.26; 0.43) | 0.29(0.20;0.39) |
| 8*df* | 0.34 (0.25; 0.42) | 0.31(0.23;0.39) |
| 9*df* | 0.35(0.27;0.44) | 0.27(0.19;0.35) |
| 10*df* | 0.36 (0.27; 0.44)] | 0.25(0.17;0.32) |
| Atrial Fibrillation |  |  |
| 3*df* | 0.33 (0.07;0.58) |  |
| 4*df* | 0.32 (0.07;0.57) |  |
| 5*df* | 0.38 (0.11;0.64) |  |
| 6*df* | 0.39 (0.12;0.65) | 0.29(0.03;0.55) |
| 7*df* | 0.40 (0.13;0.67) | 0.26(0;0.52) |
| 8*df* | 0.38 (0.11;0.66) | 0.28(0.02;0.55) |
| 9*df* | 0.42 (0.15;0.69) | 0.15(-0.12;0.43) |
| 10*df* | 0.42 (0.15;0.70) | 0.15(-0.12;0.55) |
| Coronary Heart Disease |  |  |
| 3*df* | 0.33(0.22;0.44) |  |
| 4*df* | 0.35(0.24;0.46) |  |
| 5*df* | 0.38(0.27;0.49) |  |
| 6*df* | 0.40(0.29;0.51) | 0.32(0.20;0.43) |
| 7*df* | 0.38(0.27;0.48) | 0.33(0.21;0.45) |
| 8*df* | 0.36(0.25;0.46) | 0.35(0.25;0.45) |
| 9*df* | 0.38(0.27;0.48) | 0.30(0.20;0.39) |
| 10*df* | 0.38(0.28;0.48) | 0.27(0.18;0.37) |
| Heart Failure |  |  |
| 3*df* | 0.10(-0.06;0.26) |  |
| 4*df* | 0.13(-0.03;0.29) |  |
| 5*df* | 0.17(0.01;0.24) |  |
| 6*df* | 0.17(0;0.33) | 0.08(-0.09;0.24) |
| 7*df* | 0.19(0.01;0.38) | 0.09 (-0.08;0.26) |
| 8*df* | 0.23(0.05;0.42) | 0.11 (-0.05;0.27) |
| 9*df* | 0.21(0.03;0.39) | 0.11 (-0.06;0.27) |
| 10*df* | 0.23(0.05;0.41) | 0.11 (-0.05;0.27) |
| *df*: degree of freedom | | |
